# Direct protein–protein interaction between Npas4 and IPAS mutually inhibits their critical roles in neuronal cell survival and death

**DOI:** 10.1038/s41420-021-00690-y

**Published:** 2021-10-21

**Authors:** Shuya Kasai, Xianyu Li, Satoru Torii, Ken-ichi Yasumoto, Kazuhiro Sogawa

**Affiliations:** 1 Department of Biomolecular Sciences, Graduate School of Life Sciences, Tohoku University, Aoba-ku, Sendai, 980-8578 Japan; 2 Present Address: Department of Stress Response Science, Center for Advanced Medical Research, Hirosaki University Graduate School of Medicine, 5 Zaifu-cho, Hirosaki, Japan; 3 Present Address: Department of Pathological Cell Biology, Medical Research Institute, Tokyo Medical and Dental University, 1-5-45 Yushima, Bunkyo-ku, Tokyo, 113-8510 Japan

**Keywords:** Cell death in the nervous system, Apoptosis

## Abstract

Inhibitory PAS domain protein (IPAS) is a bifunctional protein that acts as a transcriptional repressor in hypoxia and as a pro-apoptotic protein involved in neuronal cell death. Npas4 (NXF or LE-PAS) is a transcriptional factor that protects nerve cells from endogenous and foreign neurotoxins. Here we show that IPAS and Npas4 antagonize each other through their direct interaction. Coimmunoprecipitation experiments revealed that multiple binding sites on each protein were involved in the interaction. CoCl_2_ treatment of PC12 cells that induces IPAS repressed the transactivation activity of Npas4, and IPAS siRNA treatment reduced the CoCl_2_-induced repression. CoCl_2_-induced apoptosis was suppressed by the addition of KCl that induces Npas4. The protective effect of KCl was attenuated by siRNA-mediated gene silencing of Npas4. Npas4 and IPAS proteins were induced and localized in the cytoplasm of the dopaminergic neurons in the substantia nigra pars compacta after 1-methyl-4-phenyl-1,2,3,6-tetrahydropyridine (MPTP) treatment. Npas4^−/−^ mice exhibited greater sensitivity to MPTP in nigral dopaminergic neurons. Together, these results strongly suggest that neuroprotective activity of Npas4 was, at least partly, exerted by inhibiting the pro-apoptotic activity of IPAS through direct interaction.

## Introduction

Inhibitory PAS domain protein (IPAS) was originally found as a negative regulator of HIF-1 [1], a master transcriptional regulator of numerous genes under hypoxic conditions [2]. HIF-1 is composed of an oxygen-dependent HIF-1α subunit and a constitutive Arnt (HIF-1β) subunit [3, 4]. Both HIF-1α and Arnt are protein family members that are characterized by a basic helix-loop-helix (bHLH) motif and two PAS (PAS A and PAS B) domains, both of which are required for their dimerization. IPAS is a splicing variant of HIF-3α, which shows sequence similarity to HIF-1α in the bHLH and PAS domains. IPAS contains the same bHLH sequence as that of HIF-3α and one PAS A-like domain modified by alternative splicing, but lacks the PAS B domain [5]. IPAS represses HIF-1 transactivation activity by directly interacting with HIF-1α and abrogating its DNA binding activity.

We found that IPAS was upregulated by CoCl_2_-induced oxidative stress and cytokines through activation of the classical NF-κB pathway in rat pheochromocytoma PC12 cells, resulting in suppression of HIF-1 [6, 7]. Besides the transcriptional suppression function in the nucleus, IPAS could localize to mitochondria, and form a pro-apoptotic complex with Bcl-x_L_ and its related proteins in the process of CoCl_2_-induced apoptosis [8, 9]. This pro-apoptotic activity of IPAS was also demonstrated in 1-methyl-4-phenyl-1,2,3,6-tetrahydropyridine (MPTP)-induced degeneration of dopaminergic neurons in a mouse model of Parkinson’s disease (PD). Furthermore, activation of PINK1 and Parkin, both proteins are associated with early onset autosomal recessive PD, reduced IPAS-dependent apoptosis by ubiquitination and subsequent proteasomal degradation of IPAS [10].

Npas4 (also referred to as NXF or LE-PAS) also belongs to the bHLH-PAS family of transcription factors, and forms a heterodimer preferentially with Arnt2, a neuron-specific homologue of Arnt, although it is able to dimerize with Arnt [11, 12]. These dimers bind to a conserved DNA element located in the regulatory region of many target genes [13, 14]. Npas4 is found to be a neuron-specific immediate-early gene, and shows activity dependent expression [15–17]. Recent studies have revealed that Npas4 is involved in diverse neural functions such as regulation of synapses, plasticity in neural circuits, and formation of memories [18–21].

In addition, Npas4 acts as an inducible neuroprotective factor in various aspects of neurodegeneration. Npas4 was strongly induced in ischemic tissues following both focal and global cerebral ischemic insults, and this induction of Npas4 was attributed to its ability to protect neurons against toxicity of reactive oxygen species [22, 23]. Npas4 gene null (Npas4^−/−^) mice exhibited no apparent abnormality at young age. However, a small percent of them started to die when over 3 months old due to degeneration of neurons in various regions of the brain including hippocampus and cerebral cortex [13, 24]. The molecular mechanisms by which Npas4 protects neurons from a variety of neurotoxins were not fully understood. Attempts to identify subordinate genes required for protection were made [25].

In this study, we demonstrate that IPAS and Npas4 physically interact with each other, and mutually repress their biological activities in cultured cells. Transactivation activity of Npas4 was repressed by the expression of IPAS, whereas pro-apoptotic activity of IPAS was attenuated by Npas4. Nigral dopaminergic neurons were more profoundly degenerated by the administration of MPTP in Npas4^−/−^ mice. Npas4 and IPAS were simultaneously induced in the cytoplasm of SNpc dopaminergic neurons by the treatment, strongly suggesting that the neuronal cell-protective activity of Npas4 was at least partly attributed to direct interaction with IPAS.

## Results

### Interaction between IPAS and Npas4

**Fig. 1:**
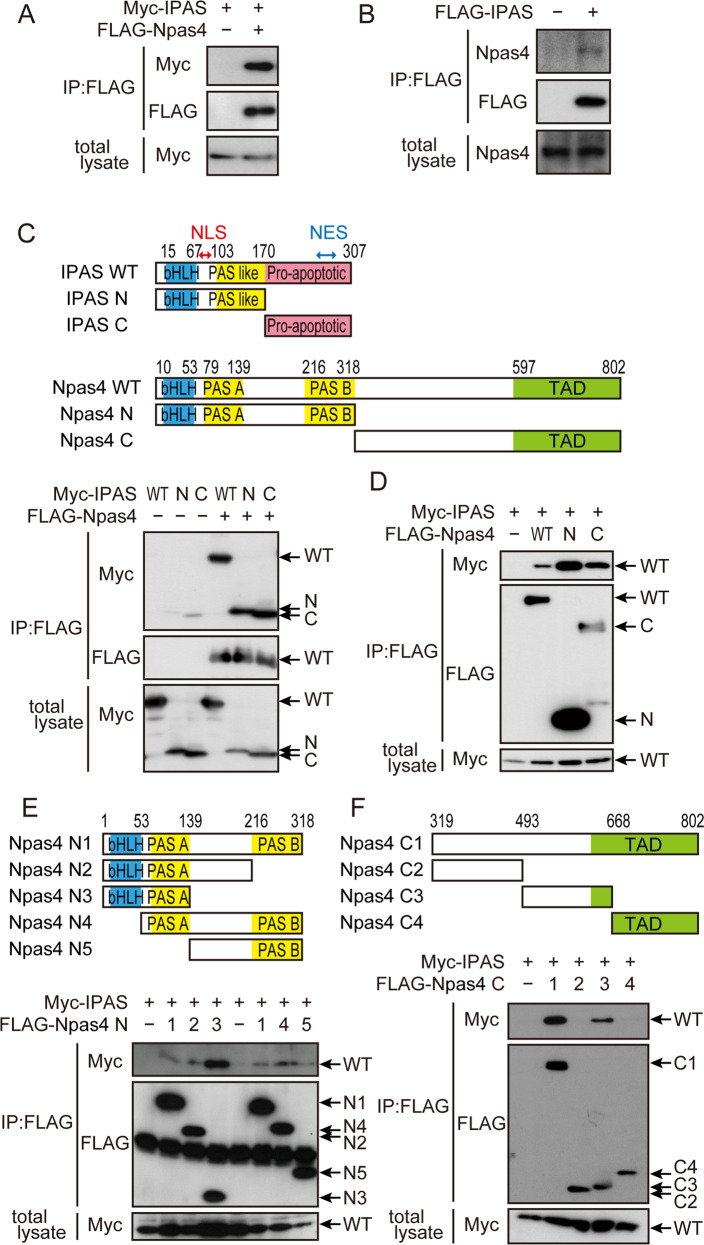
Interaction between IPAS and Npas4 by multiple sites. **A**, **B** Interaction between IPAS and Npas4. HEK293T cells were transfected with Myc-IPAS and FLAG-Npas4 (**A**) or FLAG-IPAS alone (**B**). Cellular proteins were extracted 48 h after transfection and subjected to immunoprecipitation using anti-FLAG antibody. Eluted proteins were analyzed by immunoblotting to detect Myc-IPAS (**A**) or Npas4 (**B**). **C** Schematic representation of the structure of IPAS, Npas4, and their deletions, and binding of IPAS and its deletions to Npas4. Structure of IPAS, Npas4 and their deletions is shown above. bHLH basic helix-loop-helix, NLS nuclear localization signal, NES nuclear export signal, TAD transactivation domain. HEK293T cells were transfected with indicated combinations of expression plasmids, incubated for 48 h and cell lysates were analyzed by immunoprecipitation with antibody against FLAG followed by immunoblotting with antibody against Myc. **D** Binding of IPAS to Npas4 and its deletions. HEK293T cells were transfected with indicated combinations of expression plasmids and incubated for 48 h. Cell lysates were analyzed as in (**A**). **E**, **F** Structure of deletion mutants of Npas4 used is shown above. Binding of IPAS to the deletion mutants were analyzed as shown in (**A**).

We investigated direct interaction between Myc-IPAS and FLAG-Npas4 that were overexpressed in HEK293T cells. Coimmunoprecipitation assays clearly showed that the two tagged proteins could bind to each other (Fig. 1A). Interestingly, Npas4 was expressed in non-neuronal HEK293T cells as shown in Fig. 1B. This endogenous Npas4 also interacted with FLAG-IPAS. Deletion analysis using IPAS mutants showed that Npas4-binding sites were localized in both the N- and C-terminal regions (Fig. 1C). Similarly, the binding activity of Npas4 to IPAS was localized in both the N- and C-terminal regions (Fig. 1D). Further deletions of the N-terminal segment of Npas4 strongly suggested that bHLH, PAS A, and PAS B domains, which are involved in dimerization with Arnt2, were used for binding to IPAS (Fig. 1E). The region between PAS A and PAS B showed weak repressive activity for binding. Deletion analysis of the C-terminal region demonstrated that a segment (amino acids 496-668) in the middle of the region possessed binding activity to IPAS (Fig. 1F).

**Fig. 2:**
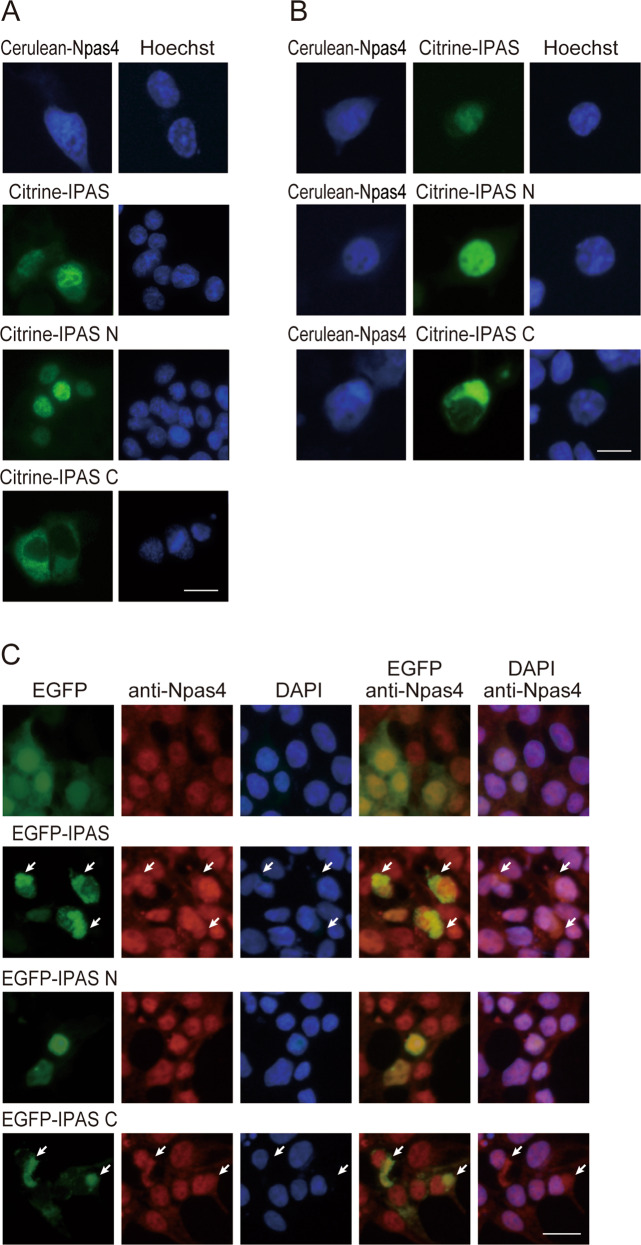
Colocalization of IPAS and Npas4 in PC12 cells. **A** Subcellular localization of Cerulean-Npas4, Citrine-IPAS, Citrine-IPAS N and C. PC12 cells were transfected with 1.6 µg of expression plasmids, and incubated with z-VAD-fmk for 24 h. Cells were stained with Hoechst 33342 for 1 h and observed using a fluorescence microscope. **B** Colocalization of Cerulean-Npas4 with Citrine-IPAS, Citrine-IPAS N and C. PC12 cells were transfected with 0.8 µg each of Cerulean-Npas4 and Citrine-IPAS, Cerulean-Npas4 and Citrine-IPAS N, and Cerulean-Npas4 and Citrine-IPAS C. 24 h after transfection, cells were observed as in (**A**). Cerulean-Npas4 was mainly colocalized with Citrine-IPAS N in the nucleus and with Citrine-IPAS C in the cytoplasm. **C** Cytoplasmic translocation of endogenous Npas4 by EGFP-IPAS C expression. HEK293T cells were transfected with 0.8 µg pEGFP-C1, pEGFP-IPAS, pEGFP-IPAS N, or pEGFP-IPAS C. Cells were fixed 24 h after transfection and stained with anti-Npas4 antibody followed by treatment with Alexa fluor 594-conjugated anti-rabbit antibody and DAPI. Two-channel images were generated by merging Npas4 and EGFP or Npas4 and DAPI. Colocalization of Npas4 and EGFP-IPAS, and Npas4 and EGFP-IPAS C in the cytoplasm was indicated with arrows. Scale bar, 20 µm.

Cerulean (a cyan variant of GFP) tagged-Npas4 (Cerulean-Npas4) showed a homogeneous distribution in transfected PC12 cells, and Citrine (a yellow variant of GFP)-IPAS also localized in both nucleus and cytoplasm (Fig. 2A). The subcellular distribution of Cerulean-Npas4 and Citrine-IPAS remained unchanged by coexpression of the two fluorescent fusion proteins (Fig. 2B). Citrine-IPAS N and Citrine-IPAS C were mainly localized in the nucleus and cytoplasm, respectively, in PC12 cells (Fig. 2A) and in HEK293T cells (Fig. 2C) as described [26]. When Cerulean-Npas4 was coexpressed with Citrine-IPAS N, Cerulean-Npas4 was predominantly localized in the nucleus together with IPAS N (Fig. 2B). In contrast, a large portion of transiently expressed Cerulean-Npas4 was colocalized with Citrine-IPAS C in the cytoplasm. Immunofluorescent staining of endogenous Npas4 in HEK293T cells showed that Npas4 was mainly localized in the nucleus (Fig. 2C). Although overexpression of EGFP or EGFP-IPAS N in the cells was unable to affect the localization of endogenous Npas4, expression of EGFP-IPAS C caused translocation of Npas4 into the cytoplasm in around 75% of cells expressing EGFP-IPAS C. Expression of EGFP-IPAS similarly induced cytoplasmic translocation of Npas4 in around 33% of cells expressing EGFP-IPAS (at least 100 cells were counted for each condition).

### Effect of IPAS expression on transactivation activity of Npas4

**Fig. 3:**
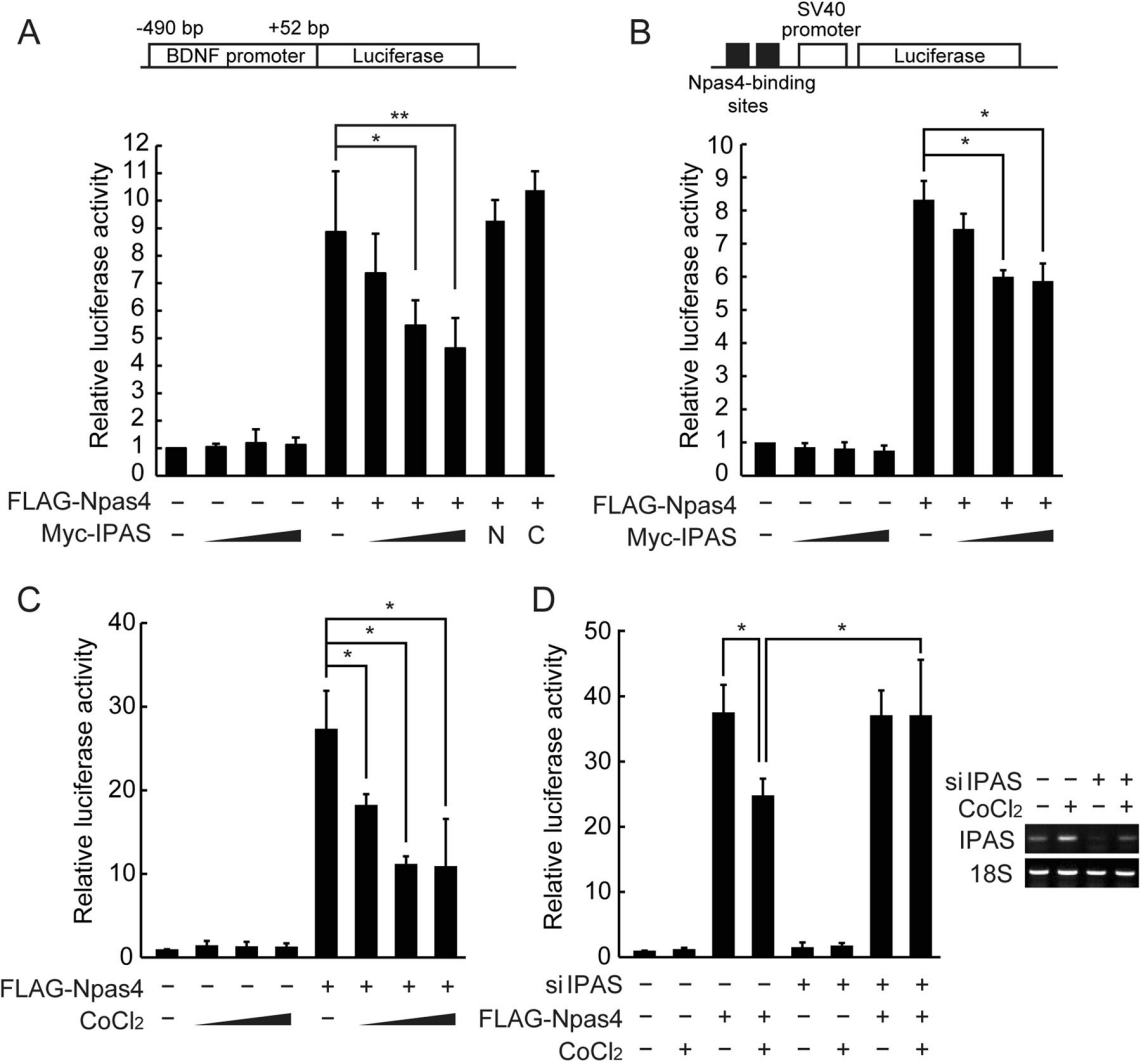
Repression of transactivation activity of Npas4 by IPAS. **A**, **B** Repression of FLAG-Npas4 transactivation activity by coexpressed Myc-IPAS. Structure of luciferase reporter with a natural BDNF promoter I (**A**) and a synthetic promoter with two Npas4-binding sites (**B**) are shown above. PC12 cells were transfected with either of reporter plasmids, the effector plasmid FLAG-Npas4 and increasing amounts of pMyc-IPAS. 24 h after transfection, luciferase activity was determined. **C**, **D** Repression of Npas4 transactivation activity by CoCl_2_-induced IPAS expression. The natural BDNF reporter plasmid was used for assays. After transfection, PC12 cells were treated with 50, 100, or 150 µM CoCl_2_ for 16 h and harvested for luciferase activity (**C**). PC12 cells were transfected with control or IPAS siRNA. Twenty-four hours after transfection, cells were transfected with the reporter and effector plasmids, incubated for 16 h in the presence of 100 µM CoCl_2_ and harvested for luciferase assay. Knockdown of IPAS mRNA expression is shown right (**D**). The experiment was performed in triplicate, and the data represent the mean ± SD. *p < 0.05; **p < 0.01.

The effect of IPAS expression on the transactivation activity of Npas4 was investigated using natural and synthetic promoters linked to the luciferase gene (Fig. 3A, B). When a luciferase reporter construct with the natural BDNF promoter I was introduced into PC12 cells together with a FLAG-Npas4 effector plasmid, luciferase activity was increased ~9.0-fold (Fig. 3A). The increased activity was repressed by the overexpression of Myc-IPAS in a dose dependent manner. Overexpression of either IPAS N or IPAS C appeared insufficient to repress the Npas4-dependent reporter activity although both truncated proteins possessed binding activity to Npas4 (Fig. 1C). Similarly, enhanced transactivation of the synthetic promoter by the expression of FLAG-Npas4 was attenuated by the coexpression of Myc-IPAS (Fig. 3B). Next, we investigated the effect of CoCl_2_ treatment, which induces IPAS as described [6] (Supplementary Fig. 1), on Npas4-induced reporter activity. The increased luciferase activity transactivated by Npas4 was reduced by CoCl_2_ treatment in a concentration-dependent manner (Fig. 3C). This repression was counteracted by the knockdown of IPAS, suggesting that transactivation activity of Npas4 was inhibited by IPAS that was induced by the addition of CoCl_2_ (Fig. 3D).

### Protection of PC12 cells by Npas4 against IPAS-induced apoptosis

**Fig. 4:**
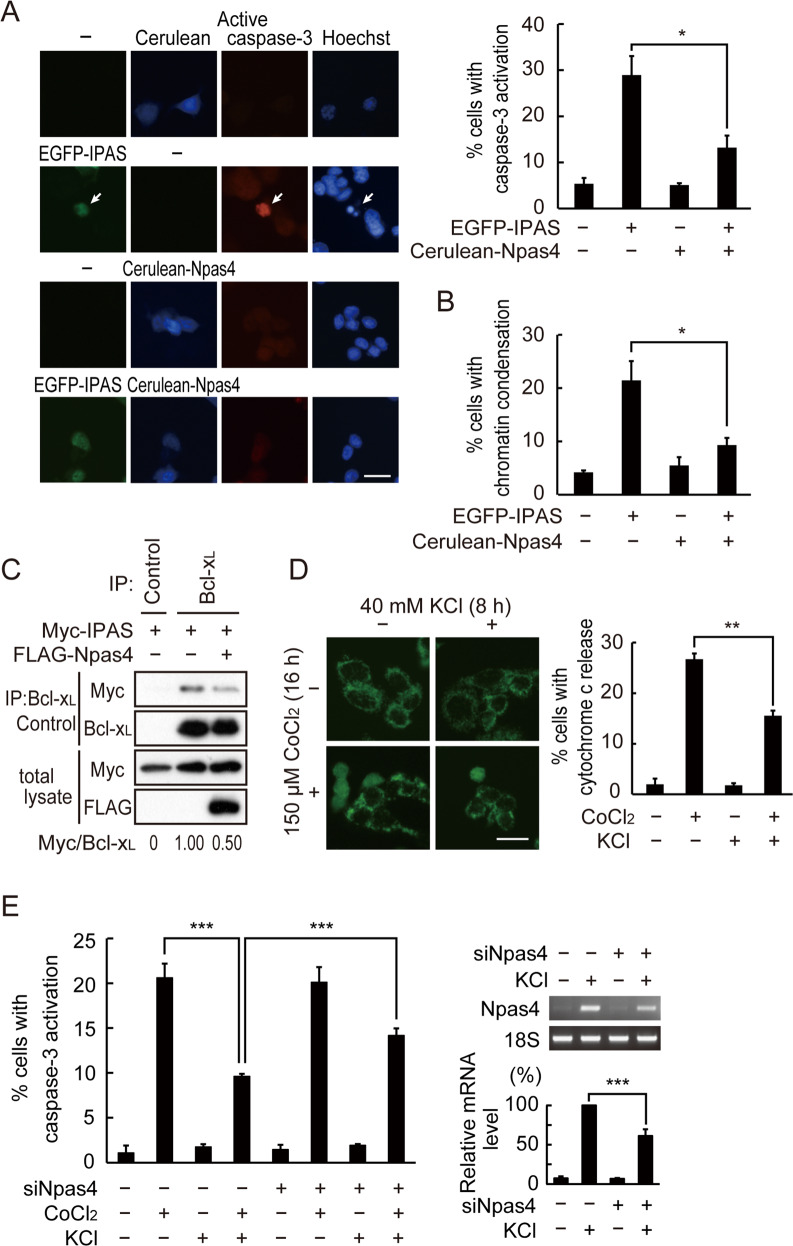
Suppression of IPAS-dependent apoptosis by expression of Npas4. **A** Inhibition of EGFP-IPAS-dependent caspase-3 activation by coexpression of Cerulean-Npas4. PC12 cells were transfected with plasmids for EGFP-IPAS or EGFP-IPAS plus Cerulean-Npas4. After incubation with z-VAD-fmk for 24 h, cells were fixed and immunostained with anti-active caspase-3. Active caspase-3-positive cells with EGFP expression were indicated by an arrow. Scale bar, 20 µm. The number of PC12 cells expressing active caspase-3 was scored. At least 300 EGFP-IPAS positive cells were counted in each experiment. **B** Inhibition of EGFP-IPAS-dependent nuclear condensation by coexpression of Cerulean-Npas4. PC12 cells were transfected as in (**A**). PC12 cells with induced nuclear condensation were scored. **C** Inhibition of interaction between Myc-IPAS and Bcl-x_L_ by FLAG-Npas4. HEK293T cells were transfected with Myc-IPAS with or without FLAG-Npas4. After incubation for 48 h, cell lysates were prepared and analyzed by immunoprecipitation with antibody against Bcl-x_L_ followed by immunoblotting with antibody against Myc. The intensity ratio of Myc-IPAS to Bcl-x_L_ was calculated and shown below. **D** Attenuation of CoCl_2_-induced cytochrome c release by KCl. PC12 cells were treated with 150 µM CoCl_2_ for 8 h and then 40 mM KCl was added. 8 h after incubation, cells were fixed and immunostained with anti-cytochrome c. Scale bar, 20 µm. Cells that show cytochrome c release were scored. **E** Suppression of CoCl_2_-induced apoptosis by KCl-induced Npas4. PC12 cells were treated with non-targeting siRNA or Npas4 siRNA for 48 h, and then treated with CoCl_2_ for 16 h and/or KCl for 8 h. After fixation and immunostained with anti-active caspase-3 antibody, active caspase-3-positive cells were counted. Npas4 knockdown efficiency was confirmed by RT-PCR (right). Three independent experiments were performed, and data are expressed as mean ± SD. *p < 0.05; **p < 0.01; ***p < 0.001.

In order to investigate the effect of Npas4 expression on the IPAS-induced apoptosis, EGFP-IPAS was coexpressed with Cerulean-Npas4 in PC12 cells. Expression of EGFP-IPAS caused caspase-3 activation and chromatin condensation in ~29% and 21%, respectively, of transfected cells (Fig. 4A and B). However, coexpression of Cerulean-Npas4 significantly reduced the proportion of cells expressing the apoptotic markers to 13% and 9.3%, respectively. Binding of Myc-IPAS to endogenous Bcl-x_L_ was examined as an indication of pro-apoptotic activity of IPAS. The binding was significantly attenuated by the expression of FLAG-Npas4 (Fig. 4C). Treatment with CoCl_2_ for 16 h caused cytochrome c release from mitochondria to the cytosol in ~27% of treated cells, but concurrent KCl treatment considerably reduced the number of cells with cytochrome c release (Fig. 4D). CoCl_2_-induced activation of caspase-3, which is induced following cytochrome c release, was also attenuated by KCl treatment, but the extent of protection was significantly reduced by siRNA treatment against Npas4 (Fig. 4E). Time-dependent induction of IPAS and Npas4 mRNAs by CoCl_2_ and KCl, respectively, was shown in Supplementary Fig. 1. Interestingly, KCl treatment of PC12 cells weakly repressed CoCl_2_-induced expression of IPAS mRNA, suggesting that another IPAS repression mechanism that is independent of Npas4 expression may be present (Supplementary Fig. 1).

### Effect of MPTP administration on Npas4^−/−^ mice

**Fig. 5:**
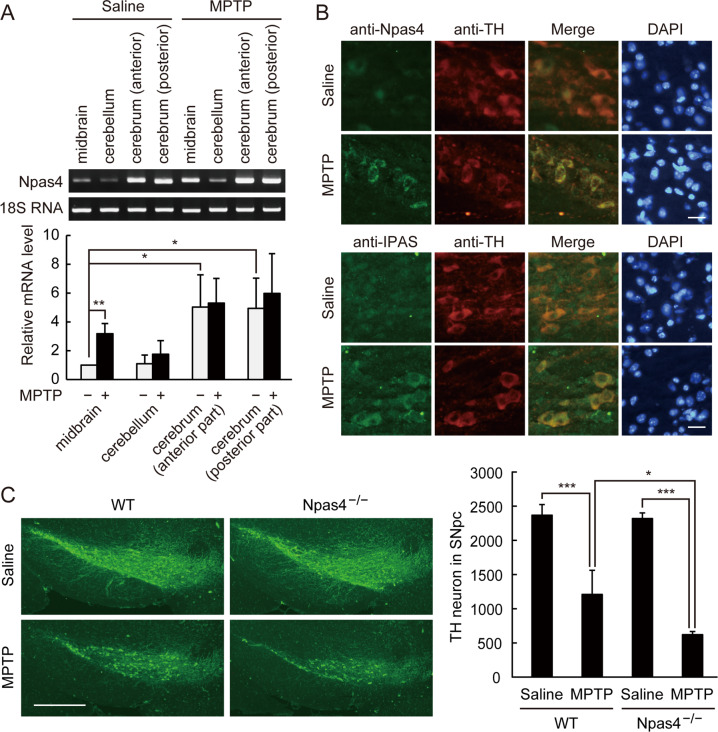
Increased toxicity of MPTP for dopaminergic neurons in the SNpc of Npas4^−/−^ mice. **A** Induction of Npas4 mRNA in the midbrain of MPTP-treated mice. Male C57BL/6J mice were intraperitoneally injected with 15 mg/kg of MPTP twice at a 2 h interval. Two hours after the second injection, total RNA was extracted from cerebrum, cerebellum, and midbrain, and Npas4 mRNA was determined by RT-PCR. Three independent experiments were performed, and data are expressed as mean ± SD. *p < 0.05; **p < 0.01. **B** Induction of Npas4 in the dopaminergic neurons of mice treated with MPTP. Mice were treated with MPTP 4 times at 2 h intervals, and brains were fixed 18 h after the final injection. Coronal sections (8 µm thick) of the SNpc were immunostained for Npas4 and IPAS. Dopaminergic neurons were immunostained with anti-tyrosine hydroxylase (TH) and were observed with a fluorescence microscope. Scale bar, 20 µm. **C** Augmented degeneration of dopaminergic neurons in the SNpc of MPTP-administered Npas4^−/−^ mice. Wild-type (WT) and Npas4^−/−^ mice were treated with MPTP 4 times as in (**B**), and killed 72 h after the final injection. Coronal serial sections of midbrain including SNpc were prepared and stained with anti-TH antibody. Scale bar, 500 µm. TH-positive neurons in every third sections (15 sections in total) through the SNpc were scored. Three independent experiments were performed, and data are expressed as mean ± SD. Statistical significance was analyzed by two-way ANOVA (genotype: *p* = 0.023, treatment: *p* < 0.001, and their interaction: *p* = 0.046) followed by post hoc Tukey–Kramer test (*p < 0.05; ***p < 0.001).

Mice were intraperitoneally injected with MPTP (15 mg/kg body weight) twice at a 2-h interval, and total RNA was extracted from the brain 2 h after the second injection. Expression of Npas4 mRNA remained unchanged in the cerebrum and cerebellum after MPTP administration. On the other hand, the expression was induced in the midbrain ~3.2-fold (Fig. 5A), although the basal expression in the midbrain was relatively low when compared with cerebrum as reported previously [27]. Npas4 protein was also found to be induced by MPTP mainly in the cytoplasm of TH-positive neurons in the SNpc (Fig. 5B). Similar to Npas4, IPAS was induced by MPTP and found in the cytoplasm.

We previously reported that IPAS expression was responsible for dopaminergic neuronal cell death in the SNpc observed in an acute MPTP mouse model of PD [10]. Using the same model, we analyzed the functional role of Npas4 in MPTP-induced neurodegeneration. Male Npas4^−/−^ mice (9–12 weeks old) were intraperitoneally administered with MPTP (15 mg/kg, 4 times, 2 h apart, the first injection was given at 10 A.M.), and TH-positive neurons in the SNpc were scored 72 h after the final injection (Fig. 5C). Administration of MPTP to control wild-type mice caused degeneration of dopaminergic neurons in the SNpc as reported, but a more profound effect on the cell killing was observed in the Npas4^−/−^ mice, suggesting the protective activity of Npas4 against the neurotoxin. Approximately 74% of dopaminergic neurons in the SNpc were lost due to MPTP toxicity in the Npas4^−/−^ mice although only 49% of neurons died by the same treatment in wild-type mice.

## Discussion

Since Npas4 has a domain arrangement similar to HIF-1α and is exclusively expressed in neuronal cells, we investigated direct interaction between IPAS and Npas4. Analysis of the interaction between the two proteins using deletion mutants strongly suggested that binding domains in Npas4 used for binding to its bHLH-PAS partners were also used for binding to IPAS. In addition, a central part in the C-terminal half of Npas4 was unexpectedly found to have the ability to bind to IPAS. On the other hand, both N- and C-terminal halves of IPAS were involved in the interaction. The C-terminal binding structure involved in binding to Bcl-x_L_ [28] may be used for binding to Npas4 because competition between Bcl-x_L_ and Npas4 for binding to IPAS was observed (Fig. 4C). This finding suggests that the C-terminal binding domain of IPAS could interact with different types of proteins with different structures.

Finding that IPAS can physically interact with Npas4 encouraged us to investigate functional consequences of the interaction because it implies that biological roles of IPAS in neuronal cell death are not only derived from interaction with Bcl-x_L_ but also from binding to Npas4. The interaction repressed transactivation activity of Npas4, and this finding demonstrates pleiotropic functions of IPAS in neuronal cell death, inhibition of cell survival activity of Bcl-x_L_ and suppression of Npas4 transactivation which plays a critical role in cell protection. Several studies reported that Npas4 possesses protective activity in neurons exposed to intracellular and extracellular stresses including excitotoxic stimulation, and the Npas4 gene is termed as one of “activity-regulated inhibitor of death” (AID) genes [15]. The neuron protective activity of Npas4 is also considered in neuronal diseases including ischemic stroke and epilepsy [22, 23, 29, 30]. In this study, it is suggested that nigral dopaminergic neurons were protected from MPTP-induced neurotoxicity in a PD model by the inducible expression of Npas4. To date, it is generally believed that the protective function of Npas4 results from its transactivation activity. A number of target genes was reported that were upregulated or downregulated by Npas4. Of these genes, BDNF and synaptotagmin 10 are selected and reported to act as protective factors against ischemia injury [31] and kainic acid-induced excitotoxicity [25], respectively. In addition to this transcription-dependent mechanism, this study strongly suggests that the protective function of Npas4 was induced by direct inhibitory interaction with IPAS, leading to reduced apoptosis of neurons. In relation to this, cytoplasmic localization of Npas4 was reported in various neurons including hippocampus and neocortex [29, 30]. Some fraction of Npas4 localized in the cytoplasm might play a role in cell survival by binding to IPAS to inhibit its pro-apoptotic activity. In addition to the neuron-protective function, Npas4 acts as a key factor involved in various neuronal functions including experience-dependent neuronal plasticity in the early developmental stage and in adult life [19, 21, 32]. Inhibitory interaction with IPAS, which is induced by hypoxic and oxidative stresses, may repress these functions of Npas4, suggesting that IPAS may mediate detrimental effects caused by hypoxia and reactive oxygen species on various neuronal activities through interaction with Npas4.

Molecular mechanism of repression of IPAS transcription by KCl is presently not known (Supplementary Fig. 1). We examined the repressive effect of Npas4 expression on IPAS promoter activity using reporter plasmids containing up to –8 kb upstream of the first ATG site of IPAS, but no transcriptional repression by Npas4 was found. This result suggests that Npas4, which is induced by KCl treatment, may not be involved in the repressive mechanism, and a more detailed analysis of the promoter region of the IPAS (HIF-3α) gene is necessary.

## Materials and methods

### Reagents and antibodies

Anti-mouse IPAS rabbit polyclonal antibody was produced as described previously [10]. Other antibodies used were obtained from the following sources: FLAG and TH from Sigma-Aldrich (St. Louis, MO, USA), Myc from MBL (Nagoya, Japan), Bcl-x_L_ from Cell Signaling Technology (Danvers, MA, USA), cytochrome c from BD Pharmingen (San Jose, CA, USA), active-caspase-3 from Promega (Fitchburg, WI, USA), and Npas4 from LSBio (Seattle, WA, USA). IPAS siRNA and control siRNA were synthesized as described [28]. ON-TARGETplus rat Npas4 siRNA and non-targeting siRNA were obtained from GE Healthcare Dharmacon (Lafayette, CO, USA). MPTP and z-VAD-fmk were obtained from Sigma-Aldrich and MBL, respectively.

### Plasmid construction

Mouse IPAS and Npas4 cDNAs were kindly provided by Drs. Y. Makino and N. Ooe, respectively. pBOS-3Myc-IPAS, pEGFP-IPAS, pCitrine-IPAS and their deletion mutants were constructed as described [10, 28]. Npas4 deletion mutants were constructed by PCR using following primers: Npas4-N4-F, 5′-CGCAG ATCTG TCTTC TTTGC TGGAG GCACT-3′; Npas4-N5-F, 5′-CGCAG ATCTG CTCTG GATGC TGATC GCCTT-3′; Npas4-N-R, 5′-CGCAG ATCTT CAGTC ACTGA TAGGG TAGTT-3′; Npas4-N2-R, 5′-CGCAG ATCTT CAGAA GAGAG AAGCA GGACC-3′; Npas4-N3-R, 5′-CGCAG ATCTT CAAGA GGGCA TGGTG AGCTG-3′; Npas4-C-F, 5′-CGCAG ATCTA CGGAA GCCTG GAGCC TCCGC-3′; Npas4-C2-R, 5′-CGCAG ATCTT CATGA GCTTT CTGTC AACTG-3′; Npas4-C3-F, 5′-CGCAG ATCTG CCAGA AGCTT TGAAG ACCAG-3′; Npas4-C3-R, 5′-CGCAG ATCTT CACAG GGGCT CCAGC CCTCC-3′; and Npas4-C4-F, 5′-CGCAG ATCTA ACCCT AACCT GTCCC TGTCA-3′. pBOS-3FLAG-Npas4 was constructed by inserting the blunt-ended *BspE* I-*Xho* I fragment of pCerulean-Npas4 into the *Hpa* I site of pBOS-3FLAG vector, and deletion mutants of pBOS-3FLAG-Npas4 were similarly constructed. The reporter plasmid containing a natural BDNF promoter was constructed by inserting a PCR-amplified fragment corresponding to the –490 to 52 region of the mouse BDNF gene into the *Kpn* I/*Xho* I site of pGL3-Basic vector using primers, BDNF-P1-F, 5′-ACGCG GTACC TCTTT CCCCT CCTAG CCTAC ACCTT TTCG-3′ and BDNF-P1-R, 5′-GCACC TCGAG GAAGA CCGCT GGGGA ACTTG TTGCT TTTC-3′. The reporter plasmid containing two Npas4-binding sites was constructed by inserting synthetic nucleotides into the *Nhe* I/*Bgl* II site of pGL3-Promoter vector. The sequences of the nucleotides are as follows; 5′-CTAGC CTAGA AATTT GTTCG TGCCA CAGAC TAGAA ATTTG TTCGT GCCAC AGA A-3′ and 5′-GATCT TCTGT GGCAC GAACA AATTT CTAGT CTGTG GCACG AACAA ATTTC TAGG-3′. All constructions were validated by sequence analysis.

### Cell culture and transfection

HEK293T and PC12 cells were obtained from the Cell Resource Center for Biomedical Research, Tohoku University, Sendai, Japan, and maintained as described previously [10, 28]. HEK293T and undifferentiated PC12 cells were transfected with plasmid DNA using Lipofectamine 2000 (Invitrogen, Carlsbad, CA, USA) according to the manufacturer’s instruction. PC12 cells were transfected with IPAS siRNA as described previously [28]. For deprivation of Npas4, PC12 cells were transfected with 20 nM ON-TARGETplus Non-targeting pool (D-001810-10-05, 5′-UGGUU UACAU GUCGA CUAA-3′; 5′-UGGUU UACAU GUUGU GUGA-3′; 5′-UGGUU UACAU GUUUU CUGA-3′; 5′-UGGUU UACAU GUUUU CCUA-3′) or ON-TARGETplus Rat Npas4 siRNA (SO-2471593G, 5′-GGUGA CAGUA UUUAC GACA-3′; 5′-GGAGA GUGUG AGCGA GCAU-3′; 5′-CAAGA ACAGC UGACG CCAA-3′; 5′-AGAAU GAGAU AGAUC GUCU-3′) using Lipofectamine RNAiMAX (Invitrogen) according to the manufacturer’s instruction. The media were replaced 4 h later, and the cells were then treated with CoCl_2_ and/or KCl 48 h after the transfection.

### RT-PCR

RNA extraction, cDNA synthesis, and PCR amplifying IPAS and 18 S rRNA were carried out as described previously [10]. Npas4 cDNA was amplified for 35 cycles with 95 °C for 30 s, 60 °C for 30 s and 72 °C for 30 s, using following primers: Npas4-exon1-F, 5′-CAGTC ATGTA CCGAT CCACC AAG-3′; Npas4-exon2-R, 5′-CACTC TCCGA CAGGT ATAGC AAC-3′.

### Immunoprecipitation and immunoblotting

HEK293T cells were transfected with various combinations of pBOS-3Myc-IPAS, pBOS-3FLAG-Npas4 and their deletion plasmids. Cell extracts were prepared 48 h after transfection and subjected to immunoprecipitation and immunoblotting as described [10]. To detect Npas4, blotted membranes were blocked with 5% nonfat dried milk in PBS, incubated with anti-Npas4 antibody diluted 1:500 in Can Get Signal immunoreaction enhancer solution 1 (Toyobo, Osaka, Japan) overnight at 4 °C, and signals were detected as described [10].

### Luciferase assay

PC12 cells were inoculated on a polyethyleneimine-coated 24-well plate at 2 × 10^5^ cells/well and cultured overnight. To assess Npas4 transactivation activity, cells were cotransfected with 0.1 µg pGL3 reporter plasmid, 0.1 µg pBOS-LacZ, 0.05 µg pBOS-3FLAG-Npas4, and 0.1, 0.3, or 0.5 µg pBOS-3Myc-IPAS (the total amount of DNA was adjusted to 0.75 µg with a pBOS empty vector). Luciferase and β-galactosidase (a control for transfection efficiency) activities were analyzed 24 h later as described previously [8]. Since CoCl_2_ inhibits β-galactosidase activity, luciferase activity of cells treated with 50, 100, or 150 µM CoCl_2_ was normalized to the protein concentration for each sample.

### Animals

Heterozygous Npas4 knockout mice (Npas4^+/−^) on a pure C57BL/6J background were kindly provided by Dr. Ooe. Male 9–12 weeks old C57BL/6J mice and Npas4-deficient mice were bred in a 12-h light/12-h dark cycle at 23 °C. C57BL/6J mice used in Fig. 5A and B were purchased from Japan SLC (Hamamatsu, Japan). Mice were intraperitoneally injected twice with 15 mg/kg MPTP or saline at a 2-h interval and sacrificed by inhalation of isoflurane 2 h after the second MPTP injection for analysis of Npas4 mRNA expression levels. Brains were removed when the animals were completely anaesthetized, and frozen in liquid nitrogen for RNA extraction. Removed brains were also fixed in formalin for immunohistochemical staining. Mice injected with MPTP (15 mg/kg) four times (2-h intervals) and sacrificed 18 h after the last injection were analyzed by immunofluorescent staining of IPAS and Npas4. Wild-type and Npas4^−/−^ littermates used in Fig. 5C were produced by crossing Npas4^+/−^ mice. Npas4^−/−^ mice used in the experiments showed no behavioral abnormality reported previously [13]. Mice injected with MPTP (15 mg/kg) four times (2-h intervals) and killed 72 h after the last injection were analyzed by counting survived TH-positive neurons in the SNpc. All animal experiments were approved by the Committee for Animal Research of Tohoku University and performed in accordance with the Regulation for Animal Experiments and Related Activities as Tohoku University (Regulation No 122).

### Immunofluorescent staining

PC12 cells were inoculated at 2.5 × 10^5^ cells/well on collagen IV-coated coverslips in a 12-well plate and cultured overnight. Cells were transfected with 1.6 µg pCitrine-IPAS, pCerulean-Npas4 or 1:1 mixture of them. Culture media were replaced 4 h after the transfection, and cells were incubated for 24 h in the presence of 10 µM z-VAD-fmk, and stained with 0.4 µg/ml Hoechst 33342. Detection of caspase-3 activation, cytochrome c release and chromatin condensation in PC12 cells were carried out as described previously [28]. HEK293T cells, inoculated 1.5 × 10^5^ cells/well on coverslips in a 12-well plate and cultured overnight, were transfected with 0.8 µg pEGFP-C1 or pEGFP-IPAS and fixed 24 h after transfection. Cells were blocked with 5% goat serum, and incubated overnight with anti-Npas4 antibody diluted 1:200 in 5% goat serum at 4 °C. After washing with PBS, cells were incubated with Alexa fluor 594-conjugated anti-rabbit antibody (1:250, Invitrogen, Carlsbad, CA, USA) and DAPI (0.4 μg/ml) at room temperature for 1 h.

Formalin-fixed paraffin-embedded brain samples were subjected to immunofluorescence double staining of IPAS and TH as described [10]. For Npas4 and TH staining, deparaffinized sections were incubated in HistoVT One (Nacalai Tesque, Kyoto, Japan) at 90 °C for 20 min and blocked with 5% goat serum in PBS. Sections were incubated with a mixture of anti-Npas4 (1:200) and anti-TH (1:2000) antibodies overnight at 4 °C and then with Alexa fluor 488-conjugated anti-rabbit, 594-conjugated anti-mouse antibodies (1:250) and 0.4 μg/ml DAPI for 1 h. Samples were mounted with Mowiol and examined using a fluorescence microscope (Olympus IX71, Olympus, Tokyo, Japan).

### Statistical analysis

Data are expressed as mean ± standard deviation of three independent experiments. Multiple comparisons were analyzed by two-way ANOVA followed by post hoc Tukey–Kramer test.

## Supplementary Information

Supplementary Information

Supplementary Figure

## Data Availability

The data supporting the findings of this study are available from the corresponding author upon request.
