# Supplementary material for: Direct protein–protein interaction between Npas4 and IPAS mutually inhibits their critical roles in neuronal cell survival and death
Source: Cell Death Discov. 2021 Oct 21;7:300. doi: 10.1038/s41420-021-00690-y (PMC8531447; doi:10.1038/s41420-021-00690-y)
Supplement: Supplementary file 1 — Supplementary Information [file 41420_2021_690_MOESM1_ESM.doc]

**Supplementary information**

**Direct protein-protein interaction between Npas4 and IPAS mutually inhibits their critical roles in neuronal cell survival and death**

Shuya Kasai, Xianyu Li, Satoru Torii, Ken-ichi Yasumoto, and Kazuhiro Sogawa

**Figure legends**

**Supplementary Fig. 1 Induction of IPAS and Npas4 mRNAs by the addition of CoCl_2_ and KCl. A** RT-PCR analysis of IPAS and Npas4 mRNAs following treatment with CoCl_2_ and/or KCl. IPAS and Npas4 mRNA levels were determined by RT-PCR using extracts from PC12 cells treated with 150 μM CoCl_2_ for 9.5, 10 and 13 h (left panel) and with 40 mM KCl for 0.5, 1 and 4 h (middle panel). Total RNA was also extracted from cells incubated with 150 μM CoCl_2_ for 9 h and then with CoCl_2_ (150 μM) and KCl (40 mM) for 0.5, 1 and 4 h (right panel). The RT-PCR products were electrophoresed on a 2% agarose gel, and representative images are shown. **B, C** Induction of IPAS and Npas4 mRNAs. Each band shown in (**A**) was quantified using the ImageJ software. Relative expression levels of IPAS (**B**) and Npas4 (**C**) mRNAs were normalized using 18S RNA as an internal standard. Data were obtained from 3 independent experiments and expressed as mean ± SD. *, p<0.05; **, p<0.01.
