# Supplementary figures and images for: Direct protein–protein interaction between Npas4 and IPAS mutually inhibits their critical roles in neuronal cell survival and death
Source: Cell Death Discov. 2021 Oct 21;7:300. doi: 10.1038/s41420-021-00690-y (PMC8531447; doi:10.1038/s41420-021-00690-y)

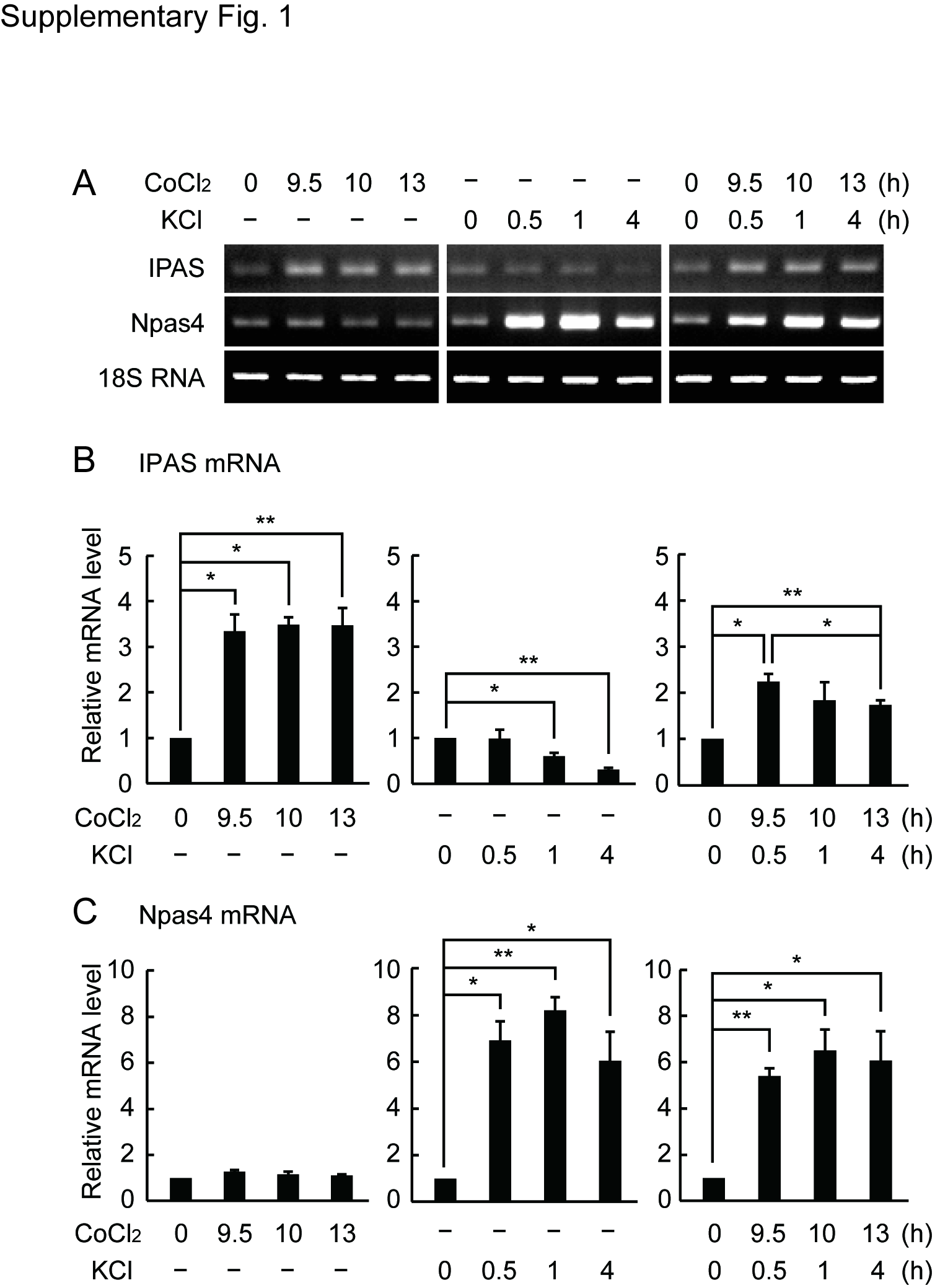

Supplement: Supplementary file 2 — Supplementary Figure [file 41420_2021_690_MOESM2_ESM.tif]
